# Prevalence of cytomegalovirus disease in kidney transplant patients in an intensive care unit

**DOI:** 10.5935/0103-507X.20170070

**Published:** 2017

**Authors:** Sanmya Danielle Rodrigues dos Santos, Antonio Tonete Bafi, Flávio Geraldo Rezende de Freitas, Luciano César Pontes de Azevedo, Flávia Ribeiro Machado

**Affiliations:** 1 Department of Anesthesiology, Pain and Intensive Therapy, Escola Paulista de Medicina, Universidade Federal de São Paulo - São Paulo (SP), Brazil.; 2 Intensive Care Unit, Hospital do Rim - São Paulo (SP), Brazil.

**Keywords:** Kidney transplantation, Cytomegalovirus, Critical illness, Immunosuppression, Intensive care units, Transplante de rim, Citomegalovírus, Doença crítica, Imunossupressão, Unidades de terapia intensiva

## Abstract

**Objectives:**

To define the frequency of cytomegalovirus disease among kidney transplant patients in an intensive care unit in which this complication was suspected and to identify predisposing factors and their possible impact on clinical outcome.

**Methods:**

Retrospective observational study in which kidney transplant patients over the age of 18 years were hospitalized for any reason in an intensive care unit with at least one collection of samples to test for the presence of antigenemia or cytomegalovirus via polymerase chain reaction during hospitalization. Cytomegalovirus disease was defined as positive antigenemia or polymerase chain reaction above 500 copies/mL in the presence of symptoms and in the appropriate clinical setting, as judged by the attending physician.

**Results:**

A total of 99 patients were included (age: 53.4 ± 12.8 years, 71.6% male). Cytomegalovirus disease was diagnosed in 39 patients (39.4%). Respiratory symptoms (51%), non-specific clinical worsening (20%) or gastrointestinal symptoms (14%) were the main reasons for exam collection. Transplant time was lower in those with cytomegalovirus disease than in those without this diagnosis (6.5 months and 31.2 months, p = 0.001), along with pulse therapy in the last 6 months (41% and 16.9%, p = 0.008) and previous use of thymoglobulin in the last year (35.9% and 6.8%, p < 0.001). In the logistic regression model, only the transplant time and the use of thymoglobulin were associated with a higher frequency of cytomegalovirus. There was no difference in clinical evolution between patients with and without cytomegalovirus disease.

**Conclusion:**

In kidney transplant patients suspected of cytomegalovirus disease, the prevalence was high. Transplant time less than 6 months, and the use of thymoglobulin in the last year should increase the intensivist's suspicion for this complication.

## Abstract (pt)

**Objetivos:**

Definir frequência de doença por citomegalovírus dentre pacientes transplantados renais na unidade de terapia intensiva nos quais houve a suspeita desta complicação; identificar fatores predisponentes e possível impacto na evolução clínica.

**Métodos:**

Estudo retrospectivo observacional, no qual foram incluídos pacientes transplantados renais acima de 18 anos, internados por quaisquer motivos em uma unidade de terapia intensiva, com pelo menos uma coleta de antigenemia ou reação em cadeia da polimerase para citomegalovírus durante internação. Doença por citomegalovírus foi definida por antigenemia positiva ou reação em cadeia da polimerase acima de 500 cópias/mL, na presença de sintomas, no contexto clínico apropriado, conforme julgamento do médico assistente.

**Resultados:**

Foram incluídos 99 pacientes (idade: 53,4 ± 12,8 anos, 71,6% homens). A doença por citomegalovírus foi diagnosticada em 39 pacientes (39,4%). Sintomas respiratórios (51%), piora clínica inespecífica (20%) ou sintomas gastrintestinais (14%) foram os principais motivos para coleta de exames. O tempo de transplante foi menor naqueles com doença por citomegalovírus em relação àqueles sem este diagnóstico (6,5 meses e 31,2 meses; p = 0,001), bem como uso de pulsoterapia nos últimos 6 meses (41% e 16,9%; p = 0,008) e uso prévio de timoglobulina no último ano (35,9% e 6,8%; p < 0,001). No modelo de regressão logística, somente o tempo de transplante e o uso de timoglobulina associaram-se à maior frequência de citomegalovírus. Não houve diferença na evolução clínica entre pacientes com ou sem doença por citomegalovírus.

**Conclusão:**

Em pacientes transplantados renais com suspeita de doença por citomegalovírus, a prevalência foi alta. O tempo de transplante menor que 6 meses e o uso de timoglobulina no último ano devem aumentar a suspeita do intensivista para esta complicação.

## INTRODUCTION

Cytomegalovirus (CMV) has the capacity to remain latent in the host cell following an acute infection.^(1)^ An imbalance between the immune system and the latent virus caused, for example, by immunosuppressive therapy, may result in viral reactivation and clinical manifestation of disease.^(2)^

Cytomegalovirus disease is a complication with high prevalence among kidney transplant patients,^(3-7)^ although there is great variation among published studies, with oscillations ranging from 5.8% to 100%.^(4-7)^ There are several reasons for this variation, but the types of populations studied and the immunosuppression^(8)^ and diagnostic test used^(7)^ are among the main reasons. Reports have shown that, in patients admitted to hospitals, the prevalence rates of infection ranged from 13.3% to 39.2%,^(3,6,9-11)^ while a study conducted in outpatients showed a prevalence of 5.8%.^(5)^ The risk factors related to CMV disease after kidney transplantation are mainly the type of immunosuppression^(8,12)^ and the serological status for CMV of the donor and recipient, with the combination of positive donor and negative recipient being characterized as the most at-risk.^(5,12)^ Cytomegalovirus leads to immune dysfunction and is associated with the risk of organ rejection; likewise, the treatment of rejection exponentially increases the risk of CMV disease,^(5,12-14)^ for which post-transplant prophylaxis or preemptive therapy regimens are used.^(3)^

All of the cited studies evaluated patients admitted to the hospital but not specifically the population of patients admitted to intensive care unit (ICU). In recent years, several studies have shown that patients admitted to the ICU and without a previous history of immunosuppression are also at risk of developing CMV disease.^(15-17)^

In this scenario, the clinical suspicion of this diagnosis in kidney transplant recipients by the intensivist in patients with clinical signs suggestive of CMV disease is important since empirical therapy is sometimes necessary because of the potential severity of the disease. Thus, we designed this study to define the prevalence of CMV disease among kidney transplant patients in the ICU with clinical suspicion of this infectious complication, to identify the predisposing factors of CMV disease and to analyze the impact of this disease on the clinical evolution of these patients.

## METHODS

This is a retrospective observational study in which data obtained from the charts of kidney transplant patients hospitalized at the *Hospital do Rim* ICU were analyzed. We included all patients over 18 years of age, with at least one collection samples to test for the presence of antigenemia for CMV via polymerase chain reaction (PCR) during the ICU stay or in the 48 hours preceding it, from September 2011 to August 2013. The request for the exams was made by the care team whenever there was clinical suspicion of CMV disease.

Patients who had graft loss more than 6 months prior and those diagnosed with CMV at admission and with a second episode of CMV disease during the same hospital stay were excluded. The study was approved by the Ethics and Research Committee of the *Escola Paulista de Medicina* of the *Universidade Federal de São Paulo* (CEP - UNIFESP), which did not consider it necessary to obtain free and informed consent, given the observational and retrospective characteristics of the study.

The relationship of the patients who had antigenemia or a CMV-positive PCR was provided by the laboratory and, from the registry number, the patients' medical records were located in the electronic system and in the medical archiving service of the hospital. Cytomegalovirus disease (CMV Group) was defined as positive antigenemia, i.e., greater than or equal to one cell, in symptomatic patients in the appropriate clinical context, according to the judgment of the attending physician and following the protocol of the service.^(18)^ CMV-positive PCRs above 500 copies/mL were also considered CMV disease, according to the service protocol.^(18)^ The remaining patients were considered to be free of CMV disease (non-CMV Group).

By means of a standardized medical record, the demographic, clinical and laboratory information related to the patient, transplant and his/her illness were collected upon hospitalization and during their ICU stay. Pulse therapy was characterized by the administration of doses greater than or equal to 500mg of methylprednisolone, according to the institutional protocol. Previous use of Thymoglobuline^®^, a rabbit anti-thymocyte globulin (thymoglobulin) was recorded in any post-transplant period, along with its use in the last 12 months. Data on the use of prophylaxis for CMV were also collected. The variables collected allowed for the calculation of the *Simplified Acute Physiology Score* (SAPS 3).^(19,20)^ The *Sequential Organ Failure Assessment* (SOFA),^(21)^ for assessing the severity of organ dysfunctions, was collected on the first day in the ICU. No additional examination was performed to enable the study. All of the information collected, including the results of the other laboratory tests, were part of the evaluation and routine care processes in the ICU. The worst values of the laboratory tests during the entire ICU stay were considered for the patients of the non-CMV Group, while in the patients of the CMV Group, only the worst exams were recorded after disease diagnosis. Sepsis was diagnosed according to the 1992 consensus criteria.^(22)^ The criteria for the diagnosis of acute respiratory distress syndrome were those of the Berlin consensus.^(23)^

The primary outcomes of interest were lethality in the ICU and in the hospital, length of stay in the ICU and hospital, time of sedation and use of vasopressors, days free of ICU and mechanical ventilation in 28 days, in addition to the presence of acute kidney injury and the need for renal replacement therapy. Acute kidney injury was defined according to the criteria Kidney Disease: Improving Global Outcomes (KDIGO),^(24)^ comparing creatinine at ICU discharge with the baseline, which was calculated based on the average of the last 3 months, disregarding hospitalizations in this period.

All patients were obligatorily followed up until they left the hospital (discharge, death or transfer).

### Statistical analysis

The categorical variables are expressed in numbers and percentages. The results of the continuous variables were expressed as the means ± standard deviations or medians (25 - 75% percentiles), according to their distribution. Distribution normality was assessed using the Shapiro-Wilk test. Categorical variables were compared using Pearson's Chi-square test and Fisher's exact test, and continuous variables were compared using the Mann-Whitney and Student's *t* tests, according to their distribution.

In the univariate analysis, those with a value of p < 0.05 were selected as potential predictors of CMV infection. They were submitted to multivariate analysis using binary logistic regression models with the *enter* method. The results of the multivariate analysis are expressed as odds ratios (OR) with 95% confidence intervals (95%CI).

All statistical calculations were performed in the Statistical Package for Social Sciences (SPSS), version 22.0. In all analyses, a two-tailed p-value < 0.05 was used as the statistical significance level.

## RESULTS

During the study period, 110 patients were subjected to the collection of material to detect antigenemia for CMV or quantification of the virus via PCR according to clinical suspicion of the care team, with no collections in the immediate postoperative period of the transplantation. Of the 110 patients, 2 were excluded because they were already being treated for CMV infection at the time of admission to the ICU and 9 because their medical records were not located, resulting in 99 patients included in the analysis. The patient characteristics are available in table 1.

**Table 1:** General characteristics of the population, according to the presence or absence of cytomegalovirus disease

Characteristics	Global (n = 99)	CMV (n = 39)	No CMV (n = 60)	p value
Age (years)	53.4 ± 12.8	53.9 ± 11.9	53.3 ± 13.4	0.820
Male	71 (71.7)	26 (66.7)	45 (75.0)	0.368
SOFA score (points)	5.2 ± 2.8	4.9 ± 2.7	5.4 ± 2.8	0.389
SAPS3 score (points)	52.1 ± 11.9	52.4 ± 12.9	51.8 ± 11.3	0.807
Source				0.198
Nursing	49 (49.5)	18 (46.2)	31 (51.7)	
Emergency room	43 (43.4)	16 (41.0)	27 (41.0)	
Surgery center	7 (7.1)	5 (12.8)	2 (3.3)	
Reason for hospitalization				0.167
Sepsis/septic shock	72 (72.7)	28 (71.8)	44 (73.3)	
Neurological	11 (11.1)	3 (7.7)	8 (13.3)	
Postoperative monitoring	8 (8.1)	6 (15.4)	2 (3.3)	
Others	8 (8.1)	2 (5.2)	6 (10.0)	
Comorbidities				
Systemic arterial hypertension	66 (66.7)	28 (71.8)	38 (63.3)	0.383
Diabetes mellitus	31 (31.3)	9 (23.1)	22 (36.7)	0.154
Coronary artery disease	6 (6.1)	3 (7.7)	3 (5.0)	0.583
Transplant data				
Kidney transplantation	94 (94.9)	37 (94.9)	57 (95)	0.977
Deceased donor	75 (75.8)	28 (71.8)	47 (78.3)	0.458
Living donor	24 (24.2)	11 (28.2)	13 (21.7)	
Double transplantation	5 (5.1)	2 (5.1)	3 (5.0)	0.977
Transplant time (months)	22.1 (5.43 - 53.3)	6.5 (1.13 - 28.2)	31.2 (14.6 - 80.7)	0.001
Transplant time				< 0.0001
< 180 days	25 (25.2)	18 (46.2)	7 (11.6)	
≥ 180 days	74 (74.7)	21 (53.8)	53 (88.3)	
Re-transplantation	6 (6.1)	2 (5.1)	4 (6.7)	0.754
Late graft function	51 (51.5)	19 (48.7)	32 (53.2)	0.653
Use of immunosuppressants				
Tacrolimus	72 (72.7)	30 (76.9)	42 (70.0)	0.450
Mycophenolate	61 (61.6)	24 (61.5)	37 (61.7)	0.990
Prednisone	94 (94.9)	37 (94.9)	57 (95.0)	0.977
Pulse therapy in the last 6 months	26 (26.3)	16 (41.0)	10 (16.9)	0.008
Prior use of thymoglobulin	32 (32.2)	18 (46.2)	14 (23.7)	0.020
In the last year	18 (18.2)	14 (35.9)	4 (6.8)	< 0.001
CMV-IgG pre-transplantation*				
Positive receiver	81 (81.8)	33 (84.6)	48 (80.0)	0.679
Positive donor	26 (52.0)	9 (50.0)	17 (53.1)	0.832
Mismatch†	3 (6.3)	1 (5.9)	2 (6.5)	0.938

CMV - cytomegalovirus; SAPS - Simplified Acute Physiological Score; SOFA - Sequential Organ Failure Assessment; CMV-IgG - IgG serology for cytomegalovirus.

* Data available for 88 recipients and 50 donors;

† Mismatch indicates positive donor and negative receiver serology. The results are expressed as numbers (%), means ± standard deviations or medians (25 - 75% percentiles).

CMV diagnostic exams were mostly motivated by respiratory symptoms (51%), new fever and nonspecific clinical worsening (20%) or symptoms of the gastrointestinal tract (14%). Thirty-nine patients (39.4%) were diagnosed with CMV (Table 2). Of these, 20 (51.2%) had positive antigenemia in the first collection, 4 (10.3%) had positive antigenemia in the second collection, and 15 (38.5%) were diagnosed by PCR. Four (10.3%) patients had positive results for both antigenemia and PCR. Ten (25.6%) patients underwent intestinal biopsy, and three (7.7%) had positive results, but two (5.1%) already had positive antigenemia and one (2.6%) had a CMV-positive PCR. Two patients had a second episode of CMV disease, but only the first episode was included in the analysis.

**Table 2:** Evolutionary characteristics of patients with and without cytomegalovirus disease

Characteristics	Global (n = 99)	CMV (n = 39)	No CMV (n = 60)	p value
Reason for clinical suspicion				0.309
Respiratory symptoms	51 (0.51)	19 (0.48)	32 (0.53)	
New picture of fever or clinical worsening	20 (0.20)	7 (0.18)	13 (0.21)	
Abdominal pain/bloating or diarrhea	14 (0.14)	8 (0.20)	6 (0.1)	
Bleeding	6 (0.06)	2 (0.05)	5 (0.08)	
Pancytopenia	2 (0.02)	1 (0.02)	1 (0.016)	
Oral ulcers	1 (0.01)	1 (0.02)	0	
Not identified	5 (0.05)	2 (0.05)	3 (0.05)	
Evolution in ICU				
Use of vasopressors	71 (71.7)	31 (79.5)	40 (66.7)	0.166
Use of mechanical ventilation	75 (75.8)	31 (79.5)	44 (73.3)	0.485
ARDS	28 (28.3)	13 (33.3)	15 (25)	0.036
Transfusion of blood products	48 (48.5)	24 (61.5)	24(40)	0.036
Laboratory changes*				
Leukocytes				0.931
Leukopenia	31 (31.3)	13 (33.3)	18 (30)	
Leukocytosis	49 (49.5)	19 (48.7)	30 (50)	
No changes	19 (19.2)	7 (17.9)	12 (20)	
Thrombocytopenia	40 (40.4)	13 (33.3)	27 (45)	0.248
LDH change	59 (83.1)	21 (87.5)	38 (80.9)	0.479
GOT change	18 (20.7)	7 (22.6)	11 (19.6)	0.746
GPT change	14 (16.1)	7 (22.6)	7 (12.5)	0.220
Change in bilirubin	20 (23.5)	9 (30)	11 (30)	0.299
Hemoglobin		7.0 [6.0 - 8.7]	7.5 [6.5 - 10.0]	0.05
Time to collect AgCMV (days)	2.2 (0- 4.0)	0 (0 - 5.0)	2.0 (1.0 - 4.0)	0.632
Treatment of CMV				
Treatment	34 (34.1)	32 (82.0)	2 (3.3)	< 0.0001
Treatment time (days)		13.1 ± 6.7	5.5 ± 0.7	< 0.0001
Outcomes				
Mortality in the ICU	65 (65.7)	26 (66.7)	39 (65)	0.865
Hospital mortality	75 (75.8)	31 (79.5)	44 (73.3)	0.485
Length of ICU stay (days)	9.0 (6.0 -18.0)	14.0 (7.0 - 31.0)	8.0 (5.25 - 16.0)	0.634
Length of hospital stay (days)	19.0 (11.0 - 38.0)	31.0 (17.0 - 53.0)	14.5 (9.25 - 30.75)	0.001
MV free time (days)	6.0 (3.0 - 22.00)	7.0 [3.0 - 22.0]	4.0 (2.0 - 20.0)	0.461
ICU free time (days)	0 (0 - 5)	0 (0 - 14.0)	0 (0 - 4.0)	0.437
Sedation time (days)	4.0 (0 - 9.0)	5.0 (1.0 - 11.0)	4.0 (0.0 - 8.0)	0.278
Time of vasopressors (days)	3.0 (0 - 7.0)	3.0 (1.0 - 7.0)	3.0 (0 - 6.7)	0.322
Renal dysfunction	70 (70)	28 (71)	42 (70)	0.848
Need for RRT				0.328
No need for RRT	25 (25.3)	7 (17.9)	18 (30)	
RRT before admission to the ICU	14 (14.1)	5 (12.8)	9 (15)	
RRT upon admission to the ICU	60 (60.6)	27 (62.9)	33 (55)	

CMV - cytomegalovirus; ICU - intensive care unit; ARDS - acute respiratory distress syndrome; LDH lactate dehydrogenase. GOT - glutamic oxalacetic transaminase. GPT - glutamic pyruvic transaminase. AgCMV - cytomegalovirus antigenemia; MV - mechanical ventilation; RRT - renal replacement therapy.

* Leukopenia ≤ 4,000 leukocytes/mm^3^; leukocytosis ≥ 12,000 leukocytes/mm^3^; thrombocytopenia ≤ 100,000 platelets/mm^3^; change in LDH ≥ 250 U/L; change in GOT ≥ 200 U/L; change in GPT ≥ 200 U/L; change in bilirubin ≥ 2.0mg/dL. The results are expressed as numbers (%), means ± standard deviations or medians (25-75% percentiles).

Regarding the serology for pre-transplantation CMV, 81 recipients (81.8%) had positive serology, whereas in donors, this percentage was 52.0% (Table 1). Although the information was not available for all patients, the serology of the donor was positive in only three situations, while that of the recipient was negative. None of the patients received prophylaxis for CMV, according to institutional practice.

Patients diagnosed with CMV did not differ from those without this diagnosis in terms of demographic characteristics, origin, reasons for hospitalization and the presence of comorbidities. However, the time of transplantation was lower in those whose CMV diagnosis was confirmed (6.5 months) than in those without this confirmation (31.2 months), with p = 0.001. The CMV Group received pulse therapy more frequently in the last 6 months than the non-CMV Group (41% *versus* 16.9%, p = 0.008). The previous use of thymoglobulin both at any post-transplant period and in the last 12 months were associated with a higher frequency of CMV diagnosis (prior use: 46.2% *versus* 23.7%, p = 0.02, the last 12 months: 35.9% *versus* 6.8%, p < 0.001) (Table 2).

The multivariate analysis showed that patients with transplant time less than 6 months were more likely to develop CMV than those with more than 6 months of transplantation (OR 4.365; 95%CI 1.497 - 12.785 p = 0.007). Likewise, those patients who used thymoglobulin in the last year also had a higher chance of CMV infection compared with those who did not use thymoglobulin (OR 4.855; 95%CI: 1.344 - 17.530; p = 0.016).

Thirty-two patients (82%) of the CMV Group were treated: 31 with ganciclovir and only 1 with foscarnet because that individual did not show clinical improvement after the use of ganciclovir. Seven patients in the CMV Group received no treatment, and six died before the results were available: five before the PCR result and one before the antigenemia result. Among them, four had received thymoglobulin in the last year and had less than 6 months of transplantation; the other three did not exhibit the risk factors found in this study. One of the survivors had a CMV-positive PCR and did not receive antiviral treatment because he showed spontaneous clinical improvement before the exam results were available. He also had less than 6 months of treatment and received thymoglobulin in the past year.

During the ICU stay, the two groups exhibited similar evolution regarding the necessity of vasopressor and the use of mechanical ventilation. Patients with CMV were more frequently diagnosed with acute respiratory distress syndrome and used more blood components (Table 2). Mortality rates, both in the ICU and in-hospital, were similar in both groups.

## DISCUSSION

Comparison of our prevalence outcome with those of other studies is complex because the populations analyzed and the criteria for defining disease or asymptomatic infection are not uniform. Studies usually consider laboratory or microbiological identification of CMV as an infection in the absence of clinical symptoms. This distinction is critical for the appropriate evaluation of the prevalence of CMV disease, characterized by the presence of CMV infection and compatible symptomatology. There are reports of similar prevalence (39.2%) using only antigenemia and the presence of symptoms,^(3)^ while studies also using increases in antibody titers, in addition to these criteria, show a lower prevalence (5.8%) among outpatients. Other studies evaluated the prevalence in patients outside the ICU, finding CMV disease in 19% and 16.6% of the transplanted patients.^(9,10)^ Diagnostic classification, whether infection or disease, also has an impact on prevalence. Evaluating a sample from the *Hospital do Rim* without differentiating these two conditions indicated a prevalence of 13%.^(6)^ The difficulty in these definitions is clearly demonstrated by a new publication from the same hospital, now with a prevalence of infection or disease of 48% in the first 3 months after transplantation, showing how time in relation to transplantation may interfere in the evaluation of prevalence.^(11)^ In our study, a higher prevalence was expected since the patients were more severe and all showed symptomatology. In addition, we used a very sensitive criterion to consider the presence of CMV disease, that is, the presence of any positive counts in the antigenemia. This finding, combined with the use of PCR, may have contributed to better detection. However, the symptoms often reported as possible CMV disease may be secondary to other bacterial, fungal, or viral infections common in ICU patients. Thus, it is possible that there were false-positives among our patients diagnosed as having CMV disease, overestimating the prevalence.

The risk factors found here, i.e., transplant time less than 6 months and use of thymoglobulin in the last year, are widely described in the literature, and knowledge of them is important to guide empirical treatment in suspected cases of infection. Although these factors are already known, among the seven patients who did not receive treatment, four had less than 6 months of transplantation and received thymoglobulin in the last year, indicating a need for medical attention. The association between transplantation time and CMV disease may be explained by the higher immunosuppressive load in the period closest to transplantation.^(4,5,7,25,26)^ We also showed that hospital mortality of critical kidney transplant patients with clinical suspicion of CMV disease was high, regardless of the laboratory evidence of this infection. This high mortality may be a consequence of the high complexity of the population admitted to the ICU, as seen by the severity scores used. In addition to the immunological disorders attributed to transplant-related immunosuppression, alterations secondary to sepsis, the main cause of admission, are added as contributing factors for the high morbidity and mortality of these patients. However, it is important to emphasize that we did not compare patients with suspected CMV with those who were not suspected to have CMV; we also did not compare patients with suspected CMV with patients without severe illness outside the ICU environment.

One of the strengths of our study is its originality. To our knowledge, there are no studies in the literature on CMV in kidney transplant patients in the ICU. The recognition of the factors related to the presence of CMV disease in severe kidney transplant patients may help the intensivist in decision making regarding empirical therapy. Another strong point is the consecutive selection, not convenience, of our sample. However, this study has several limitations. This is a retrospective database analysis with a small sample, and the study was performed in a single center. The actual prevalence of disease in ICU transplant patients cannot be defined, as there is no adequate record of the number of kidney transplant patients admitted to the ICU in the period studied, which also makes it impossible to correctly ascertain the percentage of transplanted patients with clinical suspicion of CMV. As the clinical suspicion of CMV disease was a defining factor in the decision to collect antigenemia or PCR samples, the prevalence reported here may have been overestimated. We could also not determine whether CMV disease was the main cause of ICU admission or whether the disease was found to be concomitant in patients hospitalized for other causes. Another limitation is the absence of a defined protocol for requesting the exams.

## CONCLUSION

The prevalence of cytomegalovirus disease in kidney transplant patients hospitalized in an intensive care unit is high when there is clinical suspicion of this infection. The predisposing factors independently associated with increased risk of cytomegalovirus disease in this population were transplant time less than 6 months and thymoglobulin use in the last year. Patients with cytomegalovirus disease did not present a worse clinical evolution compared to patients without cytomegalovirus.

### Contribution of each author

SDR Santos and LCP Azevedo designed the study. SDR Santos was responsible for data collection. SDR Santos, AT Bafi, FGR Freitas and FR Machado participated in the analysis of the data and the preparation of the manuscript. All authors reviewed and approved the final version of the manuscript.
